# Ultrasound-Guided External Oblique Intercostal Plane Block for Postoperative Analgesia in Laparoscopic Sleeve Gastrectomy: A Prospective, Randomized, Controlled, Patient and Observer-Blinded Study

**DOI:** 10.1007/s11695-024-07174-9

**Published:** 2024-03-18

**Authors:** Ali Sait Kavakli, Taylan Sahin, Umit Koc, Arzu Karaveli

**Affiliations:** 1 Department of Anesthesiology and Reanimation, Faculty of Medicine, Istinye University, 34396 Istanbul, Turkey; 2 Department of General Surgery, Faculty of Medicine, Istinye University, 34396 Istanbul, Turkey; 3 Department of Anesthesiology and Reanimation, University of Health Sciences, Antalya Training and Research Hospital, 07100 Antalya, Turkey; 4 Istinye Universite Hastanesi, Aşık Veysel Mah, Süleyman Demirel Cd. No:1, 34517 Esenyurt, Istanbul, Turkey

**Keywords:** Ultrasound guided, External oblique intercostal plane block, Sleeve gastrectomy, Bariatric surgery, Postoperative, Analgesia

## Abstract

**Purpose:**

The external oblique intercostal plane (EOI) block is a novel block technique for anterolateral upper abdominal wall analgesia. The superficial nature of the external oblique intercostal plane allows it to be easily identified even in patients with obesity. The aim of this study was to test the hypothesis that EOI block would reduce IV morphine consumption within 24 h after laparoscopic sleeve gastrectomy.

**Materials and Methods:**

Patients were randomly assigned to one of two groups: EOI block group and control group. The patients in the EOI block group received ultrasound-guided bilateral EOI block with a total of 40 ml 0.25% bupivacaine after anesthesia induction. The patients in the control group received no intervention. Postoperatively, all the patients were connected to an intravenous patient controlled analgesia (PCA) device containing morphine. The primary outcome of the study was IV morphine consumption in the first postoperative 24 h.

**Results:**

The median [interquartile range] morphine consumption at 24 h postoperatively was significantly lower in the EOI block group than in the control group (7.5 [3.5 to 8.5] mg vs 14 [12 to 20] mg, *p* = 0.0001, respectively). Numerical rating scale (NRS) scores at rest and during movement were lower in the EOI block group than in the control group at 2, 6, and 12 h but were similar at 24 h. No block-related complications were observed in any patients.

**Conclusion:**

The results of the current study demonstrated that bilateral EOI block reduced postoperative opioid consumption and postoperative pain in patients with obesity undergoing laparoscopic sleeve gastrectomy.

**Trial Registration:**

Clinicaltrials.gov identifier: NCT05663658.

**Graphical Abstract:**

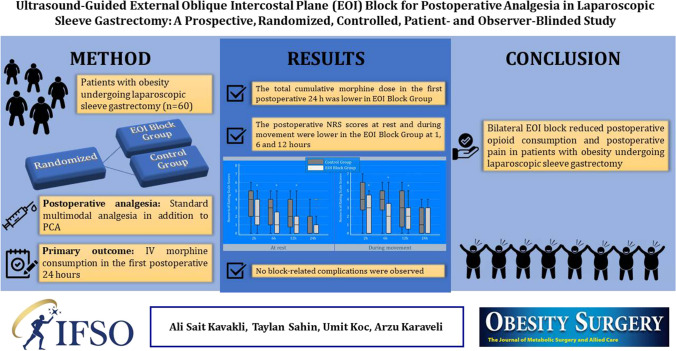

## Introduction

Postoperative pain after laparoscopic sleeve gastrectomy, caused by factors such as surgical manipulation of the stomach, surgical position, and inflation pressure, may cause immobilization and inability to perform respiratory physiotherapy, increasing the risk of postoperative complications. However, the use of opioids for postoperative pain management in patients with obesity undergoing sleeve gastrectomy is limited due to a higher incidence of obstructive sleep apnea [1]. Some regional anesthesia techniques such as transverse abdominis plane (TAP) block, quadratus lumborum (QL) block, and erector spinae plane (ESP) block may be used as a part of a multimodal analgesic regimen to reduce opioid consumption [2, 3]. However, technical challenges such as difficulty in identification of the structures and the limitation in needle movement may be experienced due to deep anatomical location of structures when performing these blocks in patients with obesity [4].

The external oblique intercostal plane (EOI) block is a novel modified block technique for anterolateral upper abdominal wall analgesia, which provides blockade of the lateral and anterior cutaneous branches of the intercostal nerves from T6/7 to T10/11 [5]. As the location is more superficial than in TAP, ESP, and QL blocks, the EOI block has the advantage of greater ease of application, especially in patients with obesity. It can also be performed in the supine position as it is distant from vascular structures, and if a catheter needs to be placed, the catheter insertion site is far from the operation site [6].

The aim of the current study was to test the hypothesis that EOI block performed in patients with obesity would reduce morphine consumption within 24 h after laparoscopic sleeve gastrectomy.

## Methods

### Study Design

Ethical approval for this prospective randomized controlled study was provided by the Ethics Committee of Istinye University, Turkey, on December 7, 2022, with approval number 3/2022.K-92. The trial was registered at ClinicalTrials.gov (identifier: NCT05663658, principal investigator: A.S.K.) on December 23, 2022, and was conducted in accordance with the principles of the Declaration of Helsinki. Written informed consent was obtained from all participants. The Consolidated Standards of Reporting Trials (CONSORT) guidelines were adhered to in reporting this study [7].

Patients aged between 18 and 65 years with an American Society of Anesthesiology (ASA) physical status score of 3 and a body mass index (BMI) > 40 kg/m^2^, who were scheduled for elective laparoscopic sleeve gastrectomy at the Istinye University Hospital, were enrolled in the study. The study exclusion criteria were defined as a history of liver or kidney disease, psychiatric disorders, alcohol or drug abuse, chronic opioid use, receipt of analgesic medication within 48 h preoperatively, history of abdominal surgery or trauma, systemic infection or local infection at the injection site, coagulation disorders, or hypersensitivity to the local anesthetics.

Patients were assigned to either the EOI block group or the control group in a 1:1 ratio using a computer-generated randomization sequence. Randomization was performed by an independent third party, and the lists were kept in numbered, sealed, opaque envelopes. The anesthesiologist performing the block was not blinded to randomization and was not involved in the data collection and evaluation processes. The patients, the anesthesiologists providing perioperative care, and the surgeon were masked to the group allocations until completion of the study. The EOI blocks were performed after induction of anesthesia to be able to guarantee the patient blindness. The observer, blinded to group allocation and responsible for data collection, remained outside the operating room during anesthesia induction and block placement and was called back to the operating room at the beginning of surgery. Outcome assessors involved in data collection were also masked to the group allocation and were not allowed access to randomization until after data analysis was complete. During the preoperative visit, an anesthesiologist who was part of the study research team instructed all the patients how to evaluate pain using a numerical rating scale (NRS) and how to use the patient-controlled analgesia (PCA) device for pain management.

### Anesthesia Management

All the patients received standardized general anesthesia. On arrival in the operating room, peripheral intravenous access was applied. The patients were monitored with 3-lead electrocardiography, pulse oximetry, and non-invasive blood pressure. General anesthesia was induced with intravenous (IV) midazolam 0.1 mg kg^−1^, propofol 2 mg kg^−1^, and rocuronium 0.6 mg kg^−1^, in addition to 100 µg of fentanyl. After intubation, the tidal volume was set as 4–6 ml/kg, and respiratory rate as 12–15 breath/min to maintain EtCO2 between 30 and 35 mmHg. Anesthesia was maintained at age-adjusted minimum alveolar concentrations (MAC) of 1.5–2 of sevoflurane in an oxygen-air mixture of 60/40%. All surgical procedures were performed by the same surgeon using the same technique. During the surgery, intra-abdominal pressure was kept at maximum 12 mmHg unless there is a problem with insufflating a “tight” abdomen. When an increase in heart rate or the mean arterial blood pressure was greater than 20% of the baseline, an additional bolus of fentanyl was given. Repeated injections of rocuronium were administered when necessary. All patients received dexamethasone 4 mg IV after induction of anesthesia and ondansetron 4 mg IV 10 min before the end of the surgery as prophylaxis against postoperative nausea and vomiting. Tramadol 100 mg and paracetamol 1 gr IV were also administered for postoperative analgesia. At the end of the surgery, all the patients received sugammadex in a dose of 2 to 4 mg kg^−1^ to reverse rocuronium. After extubation, the patients were transferred to the post-anesthesia care unit (PACU).

### Ultrasound-Guided External Oblique Intercostal Plane Block

EOI blocks were performed bilaterally after induction of general anesthesia by an experienced anesthesiologist who was excluded from the data collection. The technique described by Elsharkawy et al. [5] was used for the blocks. The patients were positioned in the supine position with the ipsilateral arm in abduction. A 5–12-MHz linear array transducer (L12-5, Philips Ultrasound Inc., Bothell, WA, USA) was positioned in a cephalad to caudad parasagittal plane at the anterior axillary line at the level of the sixth and seventh ribs in line with the xiphoid process. Using the in-plane technique, a 20-gauge 100-mm needle (Stimuplex Ultra 360, B. Braun, Melsungen, Germany) was advanced from cephalad to caudad until the tip lay in the plane between the external oblique muscle and intercostal muscles between the sixth and seventh ribs. Following hydrodissection with 2 ml of 0.9% saline to confirm the correct needle tip position, 20 ml 0.25% bupivacaine was injected. The same procedure was then repeated with 20 ml 0.25% bupivacaine on the contralateral side.

### Postoperative Analgesia Protocol

Postoperative pain was assessed using a NRS ranging from 0 (no pain) to 10 (maximum possible pain). On arrival in PACU, a protocol-trained nurse administered a bolus of 2 mg IV morphine if the NRS score was greater than 4, and this was repeated every 10 min until the NRS score was below 5. Patients were connected to the intravenous PCA device on discharge from PACU. The PCA device consisted of 0.5 mg ml^−1^ morphine and was programmed to deliver a bolus dose of 1 mg morphine only on patient demand with a 8-min lockout time and 6 mg 1-h limit. All the patients were instructed to use the PCA device only if their NRS score exceeded 4. When the Aldrete score was ≥ 9, the patients were transferred to the general surgery ward.

On arrival in the general surgery ward, all the patients were prescribed 1 gr IV paracetamol at 6-h intervals in addition to morphine PCA in accordance with the standardized multimodal analgesia protocol. If required, dexketoprofen 50 mg IV was administered for rescue analgesia, a maximum of 4 times in 24 h. Postoperative pain at rest and during movement was measured with the NRS at 2, 6, 12, and 24 h on the surgery ward by an independent staff blinded to the group allocations.

### Outcomes

The primary outcome of the study was IV morphine consumption in the first postoperative 24 h. Secondary outcomes included rescue morphine dose in PACU; NRS scores in PACU and at 1, 6, 12, and 24 h postoperatively; rescue analgesia requirement; incidence of postoperative nausea and vomiting; and block-related complications.

### Statistical Analysis

The sample size calculation was performed with the G*Power software version 3.1.9.2., based on the primary outcome of the total IV morphine consumption in the first 24 h postoperatively. A preliminary study revealed that mean ± SD IV morphine consumption in the first postoperative 24 h was 15.2 ± 7.8 in patients who underwent laparoscopic sleeve gastrectomy without EOI block. It was assumed that a reduction of at least a 50% in morphine consumption in the EOI block group compared to the control group would be clinically relevant. A *t*-test with alpha 0.05 and power 90% showed that a minimum of 24 patients per group was required to detect a statistically significant difference in 24-h IV morphine consumption. Considering potential dropouts, it was decided to include a total of 60 patients as 30 patients in each group.

Statistical analysis was performed using SPSS version 24.0 software (SPSS Inc., Chicago, IL, USA). Normality of data distribution was determined using the Shapiro–Wilk test. Normally distributed data were presented as mean (standard deviation) values and data not showing normal distribution as median (interquartile range [IQR]) values. Differences between the mean values were compared using Student’s *t*-test for normally distributed variables and the Mann Whitney *U*-test for non-normally distributed variables. Categorical variables were presented as the number (*n*) and percentage (%) of patients, and the Chi-square test and Fisher exact test were used for categorical data as appropriate. The repeated analysis of variance (ANOVA) was implemented to examine the differences between various time points. A value of *p* < 0.05 was considered statistically significant.

## Results

**Fig. 1:**
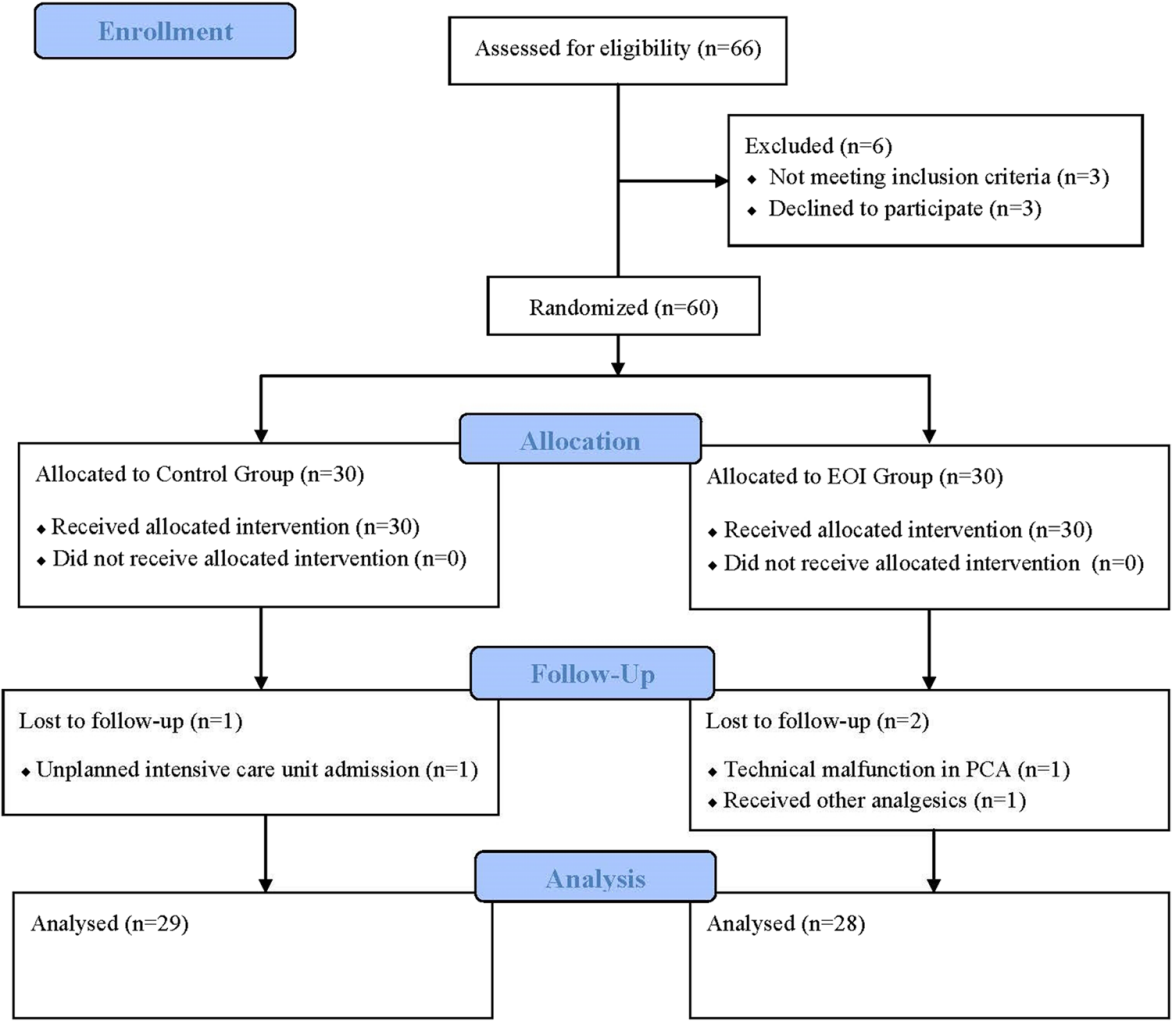
CONSORT flow diagram

From the initial assessment of a total of 66 patients for study eligibility, 3 were unwilling to participate, and 3 did not meet the inclusion criteria. Therefore, 60 patients were enrolled in the study. One patient in the control group was admitted to the intensive care unit postoperatively. In the EOI block group, postoperative pain management could not be achieved in accordance with the study protocol due to a technical malfunction in the PCA in one patient, and another patient received analgesics that were not accepted in the study protocol. The study was completed with the analysis of the data of 57 patients (Fig. 1).

The baseline patient characteristics, duration of surgery, and duration of anesthesia were seen to be comparable in both groups (Table 1). All procedures were completed laparoscopically, and no conversions to open surgery were needed.

**Table 1:** Patient characteristics (data are stated as mean (SD) or number (%))

	Control group (*n* = 29)	EOI block group (*n* = 28)	*p* value
Age, years	38 (11.7)	36.3 (12.1)	0.590
Sex, female/male	20/9 (69%/31%)	15/13 (53.6%/46.4%)	0.233
BMI, kg/m^2^	45.5 (7.2)	44.7 (5.3)	0.638
Obstructive sleep apnea	10 (34.5)	7 (25)	0.434
Duration of surgery, min	74 (14.5)	80.4 (15)	0.107
Duration of anesthesia, min	102.8 (12.8)	108.8 (13)	0.084

The total cumulative morphine dose in the first postoperative 24 h was lower in patients who received EOI block compared with the control group, representing a 46.4% reduction in the cumulative dose of morphine (median 7.5 [IQR 3.5 to 8.5] mg vs 14 [12 to 20] mg, *p* = 0.0001, respectively). Morphine was required by 15 EOI block group patients in the PACU compared to 26 patients in the control group (*p* = 0.002). Morphine consumption in the PACU was determined to be median 2 [IQR 0 to 4] mg in patients who received EOI block and 4 [2 to 4] mg in the control group (*p* = 0.007) (Table 2).

**Table 2:** Primary and secondary outcomes

	Control group^a^ (*n* = 29)	EOI block group^a^ (*n* = 28)	Difference^b^ (95% CI)	*p* value
Primary outcome				
Cumulative morphine consumption at 24 h, mg	14 [12 to 20]	7.5 [3.5 to 8.5]	− 8 (− 11 to − 6)	0.0001
Secondary outcomes				
Intraoperative fentanyl requirement, µg	100 [100 to 150]	100 [50 to 100]	0 (− 50 to 0)	0.067
Number of patients requiring morphine in the PACU	26 (89.7%)	15 (53.6%)	0.6 (0.4 to 0.9)	0.002
Cumulative morphine consumption				
PACU, mg	4 [2 to 4]	2 [0 to 4]	− 2 (− 2 to 0)	0.007
2nd h, mg	6 [5 to 8]	3 [0 to 4]	− 4 (− 5 to − 2)	0.0001
6th h, mg	11 [9 to 13]	5 [2 to 7]	− 6 (− 8 to − 4)	0.0001
12th h, mg	12 [11.75 to 17.25]	6 [2.5 to 8.5]	− 8 (− 10 to − 6)	0.0001
Postoperative nausea	12 (41.4%)	9 (32.1%)	0.8 (0.4 to 1.6)	0.470
Postoperative vomiting	6 (20.7%)	4 (14.3%)	0.7 (0.2 to 2.2)	0.529
PACU stay, min	28.8 (6.9)	26.6 (7.3)	− 2.2 (− 5.9 to 1.6)	0.251
Hospital stay, days	3.7 (0.5)	3.6 (0.5)	− 0.1 (− 0.3 to 0.2)	0.713

^a^Data are stated as mean (SD), median [IQR] values, or number (%)

^b^Data are displayed as the difference in median or mean value or relative risk, with the 95% CI

**Fig. 2:**
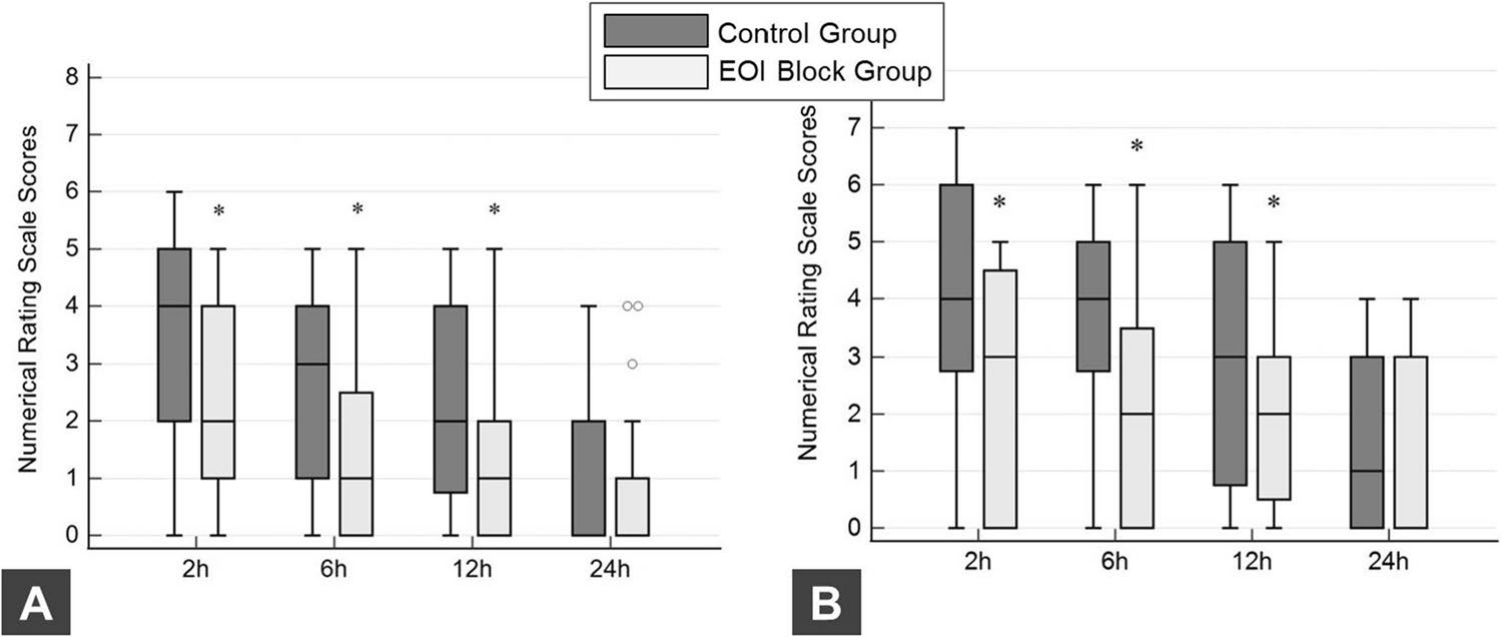
Postoperative pain scores in both groups. **A** NRS scores at rest. **B** NRS scores during movement. Box plots represent median (interquartile range), with the whiskers representing minimum and maximum values. *p < 0.05

Postoperative NRS scores at rest were significantly lower in the EOI block group than in the control group at 1, 6, and 12 h (*p* = 0.003, *p* = 0.011, *p* = 0.022, respectively). At 24 h, the NRS scores were not significantly different between the groups (*p* = 0.986) (Fig. 2A). The postoperative NRS scores during movement were significantly lower in the EOI block group than in the control group until 12 h postoperatively and were then seen to be similar in both groups at 24 h (*p* = 0.023, *p* = 0.020, *p* = 0.036, *p* = 0.899, respectively) (Fig. 2B).

There were no significant differences between the two groups in respect of postoperative nausea and vomiting (*p* = 0.360, *p* = 0.529, respectively) (Table 2). No block-related complications such as pneumothorax, insertion side bleeding, or local anesthetic toxicity were noted in any patient.

## Discussion

The results of this randomized controlled study indicated that EOI block significantly reduced the morphine consumption and pain of patients after laparoscopic sleeve gastrectomy. Cumulative morphine consumption at 24 h was reduced by a median of 46.4% with EOI block compared to patients who did not received any block, demonstrating the opioid-sparing effect of EOI block after laparoscopic sleeve gastrectomy. Pain scores at rest and during movement were significantly lower in the EOI block group compared to the control group at postoperative 12 h. The clinical significance of the difference in NRS scores in the first 12 h between the two groups is questionable because the NRS scores were less than or equal to 4 in both groups. However considering the significantly lower morphine consumption in the EOI block group, this difference in the postoperative pain scores can be attributed to the effectiveness of the EOI block.

Previous studies that have examined the efficacy of different regional anesthesia techniques such as TAP, QL, and ESP blocks in terms of postoperative pain after sleeve gastrectomy have reported significant reductions in opioid consumption [2, 3, 8]. A systematic review and meta-analysis demonstrated that TAP block improves postoperative analgesia for up to 24 h postoperatively after bariatric surgery, but the overall level of evidence was moderate to low [9]. In another study that compared the analgesic efficacy of TAP and QL blocks after sleeve gastrectomy, it was indicated that although QL block was superior to TAP block in terms of postoperative rescue analgesia, both blocks significantly relieved postoperative pain and provided comparable postoperative analgesia [2]. Elshazly et al. [3] also showed that the ESP block provided a better analgesic effect with lower postoperative opioid consumption than the TAP block after laparoscopic bariatric surgery, and stated that ESP block is more feasible than TAP block as it takes less time to perform. However, despite providing effective postoperative analgesia, some technically difficulties can be experienced when performing these blocks due to the thickness of the subcutaneous adipose tissue in patients with obesity. Although ultrasound guidance may increase the rate of block success and reduce the block-related complications, block success rates may not be as high as in patients without obesity since increased adipose tissue results in decreased quality of ultrasound images [10]. The EOI block is a superficial plane block technique, which is simple, practical, and easy to perform. The superficial nature of the external oblique intercostal plane allows it to be easily identified even in patients with obesity, and there are also the advantages of it being able to be performed with the patient in the supine position and the insertion site being far from the vascular beds and operative field [6].

To date, the postoperative efficacy of EOI block has been reported in few publications [11–13], and the literature related to patients with obesity is limited to case reports and case series [6, 14, 15]. In a retrospective cohort study, EOI block was administered to 15 of 74 patients undergoing bariatric surgery under regional anesthesia. It was reported that similar to TAP and rectus sheath blocks, the EOI block reduced opioid consumption in the first 24 h postoperatively compared to patients who did not receive any block [15]. There are also a few case reports that have demonstrated the potential utility of the EOI block in providing effective postoperative analgesia in patients with obesity [6, 14]. In the current study, both the EOI block group and control group received intravenous PCA. However, as there is no prospective comparative study in the literature comparing EOI block with no block without the use of PCA, it is difficult to test the consistency of the current study in this respect. Nevertheless, both reduced morphine consumption and lower NRS scores support the evidence that EOI block is effective in pain management after laparoscopic sleeve gastrectomy.

EOI block is a fascial plane block that can provide analgesia to the anterior and lateral abdominal wall. The limitation of the EOI block, as with other fascial plane blocks, is that it does not provide visceral analgesic coverage [5], and as with the TAP block, bilateral block may be required for midline incisions [4]. Hamilton et al. [16] stated that the external oblique intercostal plane may offer a more appropriate injection site for blocking the anterior divisions of the lateral cutaneous branches of the thoracoabdominal nerves to achieve analgesia in the anterolateral upper abdominal wall. Elsharkawy et al. [5] demonstrated the potential mechanism of the EOI block in an anatomic study showing the spread of dye injection to both lateral and anterior branches of intercostal nerves from T6/7 to T10/11. In the current study, as the EOI blocks were performed after induction of general anesthesia, the sensory dermatomal level was not assessed.

Although the opioid consumption was reduced in patients who received the EOI block in the current study, no significant difference was observed between the EOI block group and the control group in respect of the incidence of nausea and vomiting. One reason for this was that although the sample size was sufficient for the primary outcome, it may have been insufficient to evaluate the incidence of nausea and vomiting. Nausea and vomiting are reported to be associated with a significant decrease in postoperative quality of life [17] and are among the most common causes of unplanned readmission in patients undergoing bariatric surgery [18]. Although there are studies reporting the estimated incidence of postoperative nausea and vomiting up to 80% after bariatric surgery [19], it is also reported that especially high-risk patients demonstrate high satisfaction with postoperative nausea and vomiting prophylaxis [20]. Therefore, the relatively low incidence of nausea and vomiting in the current study may be related to the multimodal approach with antiemetic prophylaxis using a combination of dexamethasone and ondansetron in both groups.

No block-related complications were observed in this study. Since it is a superficial plane block technique, EOI block is likely to have a low-risk profile. The potential risks of EOI block, as for all peripheral blocks, include infection or bleeding and hematoma at the injection site and local anesthetic toxicity due to systemic uptake of local anesthetic. Due to its proximity to the lungs, the risk of pneumothorax should also not be ignored [21]. A total volume of 40 ml local anesthetic was used for bilateral EOI block in the current study as previous studies have reported using a total of 30 to 40 ml of local anesthetic for bilateral EOI block [5, 6, 11, 14]. No signs of local anesthetic systemic toxicity were observed at this dose. In the current study, the mean hospital stay was approximately 3.5 days in both groups. While some centers report mean hospital stays of one or two nights, there have also been studies reporting similar hospital stays to our findings [22]. Routine intra-abdominal drain use after sleeve gastrectomy is reported to be approximately 35% [23]. In our institution, intra-abdominal drain is routinely used after sleeve gastrectomy for 48 h, and patients are discharged from the hospital approximately 1 day after drain removal. Therefore, we believe that the prolonged hospital stay compared to previous studies may be related to this.

There were several limitations to the current study. First, because the EOI blocks were performed after induction of general anesthesia, the dermatomal level of the block was not assessed. Therefore, there is the possibility that some blocks may not have been fully effective, but considering that many peripheral blocks are performed under general anesthesia, our practice is consistent with routine clinical practice. Second, while a significant proportion of the beneficial effects of local anesthetics are achieved through absorption, a modest but not insignificant proportion is attributed to systemic analgesia [24]. In the current study, the control group did not receive any local anesthetic, including local infiltration of the port sites, whereas the EOI block group received local anesthetic, which will have had some systemic effects. Considering the statistical difference in postoperative NRS scores between the two groups, the systemic effects could be responsible for part of this. Finally, the current study revealed that a 46.4% decrease in IV morphine consumption in the first postoperative 24 h in EOI block group compared to control group. This was lower than our expectation given the assumption that a reduction in morphine consumption of at least 50% in the EOI block group compared to the control group would be clinically significant. However, the possibility that a larger population may yield more positive results in terms of postoperative opioid consumption should not be ruled out.

## Conclusion

The results of this study demonstrated that bilateral EOI block reduced postoperative opioid consumption and postoperative pain in patients with obesity undergoing laparoscopic sleeve gastrectomy. As this is the first randomized controlled study in this context, there is a need for further research to support the conclusions of this study.
